# Inflated effect estimates for vitamin D supplementation are driven by common meta-analytical errors

**DOI:** 10.1080/15502783.2024.2413668

**Published:** 2024-10-07

**Authors:** Eric T. Trexler

**Affiliations:** Duke University, Department of Evolutionary Anthropology, Durham, North Carolina, USA

**Keywords:** Vitamin D, meta-analysis, effect size

## Abstract

**Purpose:**

Han et al. (J Int Soc Sports Nutr 16:55, 2019) sought to quantify the effects of vitamin D supplementation on strength outcomes among athletes in a meta-analysis. The authors reported a pooled effect size (standardized mean difference; SMD) of -0.75 (95% CI: -1.82 to 0.32, p = 0.17) in favor of supplementation, but the analytical approach was not appropriate for a pooled analysis of randomized controlled trials and the effect sizes were calculated incorrectly. This letter discusses how these issues impact the results and interpretation of the paper, then provides an update on the estimated average effect of vitamin D on strength outcomes in athletes.

**Methods:**

Identified errors included the use of within-group rather than between-group effect size metrics, the use of standard error values in place of standard deviations, and failure to account for correlated observations within the model. The data were reanalyzed after correcting for these common meta-analytic errors.

**Results:**

The results of this reanalysis reflect a dramatically smaller and statistically nonsignificant pooled effect estimate of SMD = 0.16 (-0.24 to 0.56, p = 0.43) in favor of supplementation. Further, the model from this reanalysis has more favorable statistical characteristics than the original analysis, as evidenced by a fairly symmetrical funnel plot and a nonsignificant result for Cochrane’s Q test (Q = 5.02, p = 0.41).

**Conclusion:**

In order to disseminate robust information to sports nutrition practitioners and researchers, it is critically important for meta-analyses to produce valid effect estimates that are appropriate for the underlying study designs and calculated without error. This letter highlights common errors to inform the calculation and interpretation of future meta-analyses in sports nutrition.

Dear Editor,

An article called “Effects of vitamin D3 supplementation on serum 25(OH)D concentration and strength in athletes: a systematic review and meta-analysis of randomized controlled trials” was published in the Journal of the International Society of Sports Nutrition in November of 2019 [1]. According to publicly visible metrics on the journal website, it has been accessed over 10,000 times as of 8 June 2024. The authors should be commended for studying an important and practically applicable research topic. However, it is important to highlight key errors in the analytical approach to prevent similar errors in future meta-analyses.

In a recent review paper, Kadlec and colleagues [2] summarized a number of errors that are commonly observed in meta-analyses within the field of strength and conditioning. Sports nutrition meta-analyses often contain many of the same errors, and the presently discussed article is impacted by multiple errors. One such error is emphasizing within-group effects rather than between-group effects. The inclusion of a control group is a defining feature of the randomized controlled trial, which yields more robust findings by accounting for effects that are unattributable to the intervention itself, such as placebo effects or the effect of time.

By exclusively utilizing data from the intervention groups and omitting data from the placebo groups, the presently discussed study implements an analytic approach that fails to match the design of the studies included in the analysis. For example, the authors excluded non-randomized trials from the analysis, but this decision is only justifiable when data from the control group are actually included in the analysis. This error, which introduces the risk of erroneously inflated effect sizes and reduced p-values, was also made in a 2019 meta-analysis by Siddique and colleagues [3]. The error was rectified by retracting the original paper and republishing an updated draft with a more appropriate analysis that calculated effects sizes based on the mean difference between the intervention and control groups rather than longitudinal differences within the intervention group alone [4].

A second common error involves calculating standardized mean difference effect sizes using the standard error rather than the standard deviation in the denominator of the equation [2]. In the presently discussed meta-analysis, Han and colleagues calculated an effect size (standardized mean difference; SMD) of 3.55 for the study by Jung et al. [5], which is far larger than the range of effect sizes observed for the most efficacious dietary supplements used for the promotion of strength performance. When correcting this erroneous calculation by incorporating data from the placebo group scaling the effect size metric to pooled baseline standard deviation values instead of standard errors, the effect size drops from 3.55 to 0.52, which dramatically alters this small meta-analysis with very few studies.

While these errors may seem minor in nature, they can dramatically impact the pooled effect estimate and practical conclusions of a meta-analysis. As shown in Figure 1, I have reanalyzed the data from this meta-analysis while ensuring that all effect sizes were calculated using the change score difference between the intervention and placebo groups in the numerator, with all effect sizes standardized according to the pooled baseline standard deviation in the denominator. I further corrected the analysis by manually adjusting the placebo group sample size from the study by Close et al. [6] to avoid “double-counting” data from this group [7], although this could also be defensibly corrected by calculating a single aggregated effect size for the study or by using multi-level modeling to account for correlated data points within the meta-analysis model. The results of this reanalysis reflect a dramatically smaller and statistically nonsignificant pooled effect estimate. Further, the model from this reanalysis has more favorable statistical characteristics than the model reported by Han et al., as evidenced by a fairly symmetrical funnel plot (Figure 1b) and a nonsignificant result for Cochrane’s Q test (Q = 5.02, *p* = 0.4139).

**Figure 1.**
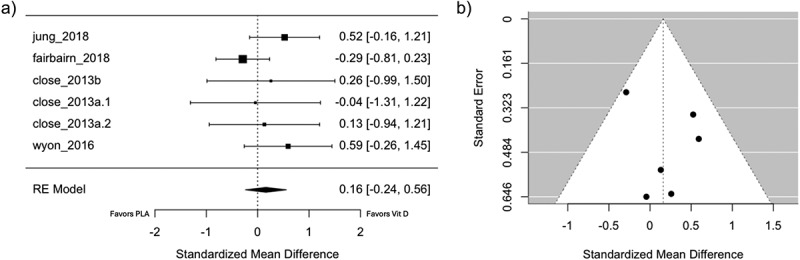
Reproduction of a) forest plot and b) funnel plot after correcting effect size calculations.

Han and colleagues recently published an updated version of this meta-analysis [8]. After including new studies on the topic and correcting the analytical errors described in this letter, the updated meta-analysis yielded a pooled effect size of SMD = 0.18 (95% confidence interval: −0.02 to 0.37; *p* = 0.08), which is extremely similar to the results presented in Figure 1. Han and colleagues should be commended for avoiding the previously listed errors in their updated analysis, but it is important to clearly identify and thoroughly describe these errors to ensure that these common mistakes are avoided by future meta-analysts, and to ensure that the effect size estimate in the present article (SMD = 0.75) is not used to plan future studies or guide the decisions of practitioners who work with athletes. Systematic reviews and meta-analyses hold a significant place in evidence-based practice and have historically been regarded as the most valid and least biased forms of scientific evidence in the evidence hierarchy [9]. In order to disseminate robust information to practitioners and researchers, it is critically important for meta-analyses to produce valid effect estimates that are appropriate for the underlying study designs and calculated without error.
